# A new species of *Lathrolestes* Förster (Hymenoptera: Ichneumonidae) from Peruvian Amazonia

**DOI:** 10.3897/BDJ.3.e4327

**Published:** 2015-03-17

**Authors:** Alexey Reshchikov

**Affiliations:** ‡ Swedish Museum of Natural History, Stockholm, Sweden

**Keywords:** Ctenopelmatinae, Perilissini, *Lathrolestes fiedleri*, South America, Neotropical region, Ucayali

## Abstract

*Lathrolestes fiedleri* **sp. n.** is described from Peru. This is the first record of the tribe and the genus for the country.

## Introduction

The subfamily Ctenopelmatinae (Hymenoptera, Ichneumonidae) is poorly represented in the Neotropical region with only 20 of 106 extant genera recorded (Reshchikov 2010, Yu et al. 2012). In total, there are only 85 described species of Ctenopelmatinae from the Neotropical region (Yu et al. 2012, Reshchikov et al. 2012). It was speculated that the group is rather scarce there due to the rarity of hosts, sawflies (Gauld 1987, Gauld et al. 1997, Veijalainen et al. 2013). The hosts of most of Neotropical species are not known (*Physotarsus adriani* Gauld, 1997 have been reared from *Dielocerus lobatus* (Erichson 1848) (Hymenoptera, Argidae) (Gauld et al. 1997), but they most likely attack tenthredinid sawflies as is known in most of the genera of Ctenopelmatinae (Kasparyan 1981, Gauld et al. 1997). Some *Lathrolestes* Förster 1869 species, *Physotarsus* Townes, 1966 and *Scolobates* Gravenhorst 1829 have been reared from Argidae (Hymenoptera) (Pschorn-Walcher H 1965, Schedl and Pschorn-Walcher 1984, Gauld et al. 1997, Zhaurova and Wharton. 2009, Reshchikov 2012). Mostly large bodied species of genera like *Himerta* Förster 1869, *Opheltes* Holmgren 1859, *Perispuda* Förster 1869, *Phobetes* Förster 1869, *Protarchus* Förster 1869, *Rhorus* Förster 1869 have been reared from Cimbicidae (Hymenoptera) (van Burst 1918, Hinz 1961, Barron 1986, Aubert 2000, Sheng et al. 2004). Members of the tribe Ctenopelmatini seem to be restricted to attack web-spinning sawflies of the family Pamphiliidae (Barron 1981, Kasparyan 2004). Australian tribe Westwoodiini is associated with Pergidae (Wharton et al. 2010, Wharton et al. 2008). Few species of *Lathrolestes* have been reared from Eriocraniidae (Lepidoptera) (Heath 1961, Barron 1994) and Megalopodidae (Coleoptera) (Barron 1994). Most of Neotropical Ctenopelmatinae biodiversity is known from the Northern part of the Neotropical region: Costa Rica (Gauld et al. 1997) and Mexico (Reshchikov 2011), but Ctenopelmatinae has not been recorded from most of the South American countries (Yu et al. 2012).

The purpose of this paper is to describe a new species of *Lathrolestes* from the Peruvian Amazonia. *Lathrolestes* belongs to Perilissini, one of the nine tribes of the subfamily Ctenopelmatinae. The genus includes 102 species [Yu and Horstmann 1997 (Yu et al. 2012 is not cited here since *L. pleuralis* (Thomson, 1883) was placed as synonym of *L. caudatus* (Thomson, 1883) by mistake in the newest version of the catalogue), Reshchikov 2013a, Reshchikov 2013b]. Nine species are known from the Northern part of Neotropical region: four species from Costa Rica (Gauld et al. 1997), four from Mexico (Reshchikov 2011) and one from Ecuador (Reshchikov et al. 2012). The new species, *Lathrolestes fiedleri* **sp.n.,** was collected in the Ucayali Region of Peruvian Amazonia. This represents the first genus-level record of the subfamily Ctenopelmatinae from Peru (Veijalainen et al. 2013) and the second record of the genus *Lathrolestes* in South America (Reshchikov et al. 2012). An illustrated diagnosis with comparisons with other Neotropical species is provided.​

## Materials and methods

*Lathrolestes* is a rarely collected genus of Ctenopelmatinae, and it is generally poorly represented in collections. This study is based on 1 specimen which was found in the MUSM.​ The morphological terminology used in this study follows that of Gauld et al. (Gauld et al. 1997). Photographs were taken with a Canon Digital Camera 5D, combined with Zerene ®.

## Taxon treatments

### Lathrolestes

Förster, 1869

Lathrolestes Förster, 1869: 196. Type-species: *Tryphon clypeatus* Zetterstedt.
Camporychus Förster, 1869. Type-species: *Lathrolestes marginatus* Thomson;
Culmina Benoit, 1955. Type-species: *Culmina ruwenzorica* Benoit;
Ecclinops Förster, 1869. Type-species: *Tryphon orbitalis* Gravenhorst;
Homalomma Förster, 1869. Type-species: *Homalomma caliroae* Rohwer;
Laphyroscopus Förster, 1869. Type-species: *Tryphon gorskii* Ratzeburg;
Lathrolestus Thomson, 1883. Type-species: *Lathrolestus clypeatus* Zetterstedt
Luphyroscopus Thomson, 1883. Type-species: *Luphyroscopus gorskii* Ratzeburg
Ritzemabosia Smits van Burgst, 1912. Type-species: *Ritzemabosia meridionalis* Smits van Burgst;
Tryphonopsis Brauns, 1898. *Tryphonopsis ensator* Brauns
Lathrolestes Tryphon clypeatus Zetterstedt, 1838

#### Diagnosis

Small to medium sized species, 4.0–7.5 mm. Occipital carina not intercepting hypostomal carina. Clypeus profile almost always flat, its apical margin thick. Head behind eyes usually narrowed. Mandibles with lower tooth distinctly longer than the upper. Pronotum with epomia absent or vestigial, never discernible as a long crescentic ridge. Epicnemial carina never reaching the fore margin of mesopleuron. Notch between postscutellum and propodeum V-shaped. Radius intercepting pterostigma at its middle or before its middle but never at its base. Areolet petiolate, oblique, sometimes absent. 2m-cu with a single bulla. Hind wing with cu-a intercepted below or at its middle. Tarsi not swollen. Tarsal claws pectinate, with basal lobe, or not pectinate. Glymmae deep. Epipleura of second and third metasomal tregites separate from tergites. Apex of subgenital plate of male not incurved on hind margin. Tip of aedeagus somewhat decurved and swollen, its apex rounded. Ovipositor sheath 0.3 to 15 as long as metasomal height. Ovipositor usully stout at base, with notch or nodus at appex but never nidle-like.

### Lathrolestes fiedleri

Reshchikov sp. n.

urn:lsid:zoobank.org:act:9120C30E-6EA8-4EED-B25A-7F95F86BD203

#### Materials

- **Type status:** Holotype. **Occurrence:** recordedBy: B. Medina; sex: female; **Location:** higherGeography: South America; country: Peru; stateProvince: Ucayali; county: Coronel Portillo; municipality: Callería District; verbatimElevation: 268m; verbatimLatitude: 08°17'34.5''S; verbatimLongitude: 73°40'52.9''W; **Event:** eventDate: 25.x.2012; **Record Level:** institutionCode: MUZM

#### Description

Body length 7 mm. Antenna with 20 flagellomeres. Scape 1.3 times as long as wide. Head narrowed behind compound eyes (Fig. 1a), matt, not punctate, shagrined. Maximum length of gena 0.63X transverse eye diameter; minimum length of gena 0.4X transverse eye diameter. Width of face equal to height of eye (Fig. 1b). In lateral profile face slightly convex, with bulge, lateral parts at inner eye margin with slight impression. Interspace between hind half of lateral ocellus and eye and vertex matt or dimly shining, 1.6X transverse ocellus diameter (Fig. 1a). Clypeus rather long, 0.5 times as long as medially wide, separated from face by deep impression (Fig. 1b). Tentorial pit not large, roundish. Malar space 1.2X basal mandible width. Mandible teeth equal. Occipital carina medially complete.

Mesosoma smooth, polished, without punctures. Notaulus not impressed. Epicnemial carina moderately high, reaching half of mesopleuron height. Hind tibia compressed. Claws elongate, not pectinate. Hind tarsus as long as hind tibia. Vein 3rs-m vestigial. Fore wing with cu-a strongly postfurcal. Propodeal carinae complete, strongly raised; area superomedia half as long as wide, anterior part (before costula) of area apicalis slightly longer than posterior part (Fig. 1c).

Metasoma compressed apically, polished, sparsely pubescent. First metasomal tergite as long as apically wide; without shallow median longitudinal impression and lateromedian longitudinal carinae, slightly curved at spiracles; with slightly enlarged epipleurae (Fig. 1d). Second metasomal tergite transverse. Subgenital plate slightly notched at apical margin. Ovipositor straight, thin, stout at base, slightly up-curved, approximately as long as metasomal height, without notch.

**Figure 2.**
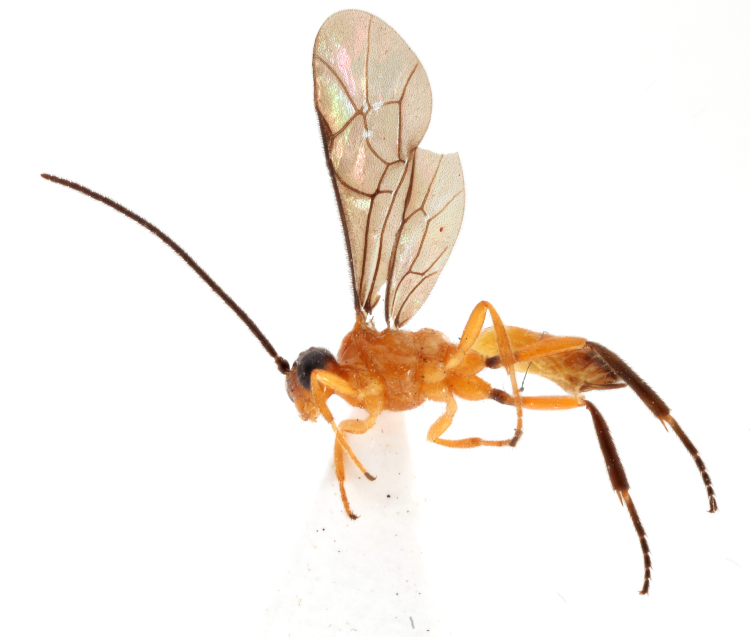
Habitus holotype female

Coloration. Female. Body mostly reddish (Fig. 2), excluding propodeum (Fig. 1c) and basal part of first metasomal tergite (Fig. 1d) pale yellowish and upper part of head (Fig. 1a), antennae, wing costae and pterostigma, hind trochanters, hind tibia and tarsus, upper part of three posterior metasomal tergites, and ovipositor sheath black (Figs 1, 2). Wings slightly infuscate.

#### Diagnosis

This species differs from other species of the genus by the combination of the following character states: elongate clypeus, 0.5 times as long as medially wide, separated from face by deep groove (Fig. 1b) (in other members of the genus clypeus shorter, 0.2-0.4 times as long as medially wide, except *L. gauldi* Reshchikov et al, 2012 and *L. protenus* Barron, 1994, species with also elongated clypeus, separated from face by deep groove, but *L. protenus* Barron, 1994 has outstanding unique character state, occipital carina intercepting hypostomal carina before base of mandible (Barron 1994)); short first metasomal tergite, as long as apically wide (in other members of the genus 1.2 - 1.8 times as long as apically wide, excluding *L. zeugophorae* Barron, 1994 and *L. gauldi*, but *L. zeugophorae* characterized by shorter clypeus and lack of costula) without longitudinal dorsal carinae (Fig. 1d); short area superomedia, 0.5 times as long as broad (Fig. 1c); infuscate wings (Fig. 2) with resemblance of areolet; and claws elongate, not pectinate. This species is morphologically closely related to *L. gauldi* and can be grouped together by the combination of the character states mentioned above. Synapomorphies shared with *L. gauldi* are short area superomedia (0.5 times as long as broad), infuscate wings, vestigial areolet, and elongate, not pectinate claws. The new species clearly differs from the Ecuadorean species in coloration (reddish face, gena, hind coxa and femur, and metasomal tergites except the last three tergites; Figs 1, 2), and by the lack of longitudinal dorsal carinae of first metasomal tergite. *Lathrolestes gauldi* has a black face, gena, completely black hind legs and metasoma, and defined longitudinal dorsal carinae of the first metasomal tergite (see Reshchikov et al. 2012).

#### Etymology

This species is named after Arkady Fiedler.

#### Distribution

Peru.

#### Conservation

This species seems to be a rare species, and further sampling is needed to clarify its distribution in the Western Amazonia.

## Discussion

In the summer of 2014, while participating in the 8th International Congress of Hymenopterists in Cusco, I had the opportunity to go through the collections of Cusco University and Museo de Historia Natural de la Universidad Nacional Mayor de San Marcos (MUSM). I also did some collecting in South Eastern Peru in August 2014. Previously I had checked the Neotropical Ichneumonidae collections of several other institutions (ANSP, AMNH, NHRS, USNM, ZMUT). Despite all my efforts and the large sampling effort taking place in many Amazonian study localities (Veijalainen et al. 2013) I encountered the first and so far only *L. fiedleri* **sp. n.** specimen in the MUSM collection. Similar to the previousely described Amazonian species, *L. gauldi* Reshchikov et al 2012, *L. fiedleri* **sp. n.** seems to be a rare species, and further sampling is needed to clarify its distribution in the Western Amazonia. I consider the description of this species as rather important for drawing attention to the loss of biodiversity in the region due to mining and logging (Gardner 2012, Goncalves et al. 2012, Risen 2011, Swenson 2011).

## Supplementary Material

XML Treatment for Lathrolestes

XML Treatment for Lathrolestes fiedleri

## Figures and Tables

**Figure 1a.**
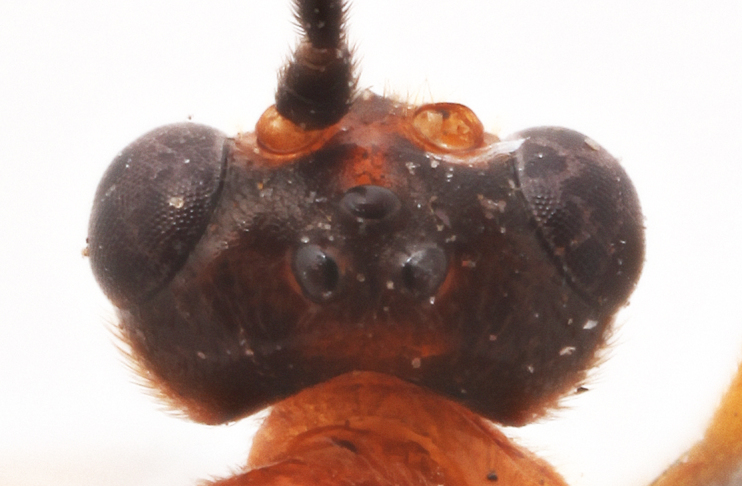
Face

**Figure 1b.**
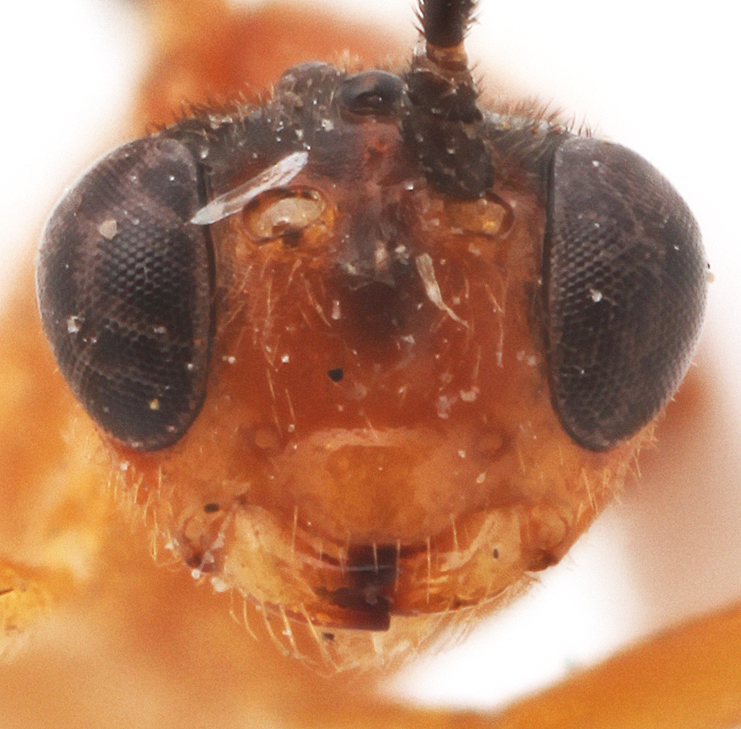
Head

**Figure 1c.**
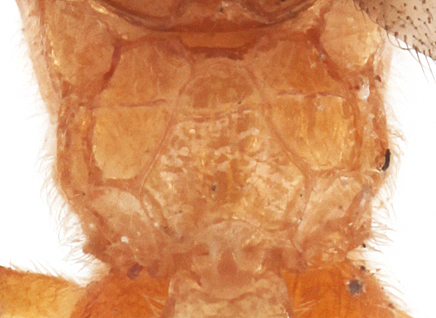
Propodeum

**Figure 1d.**
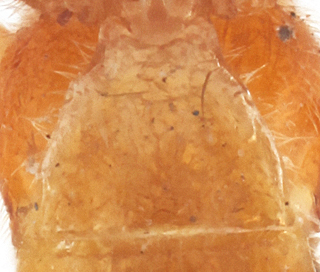
First metasomal tergite
